# Modulation of MAPK- and PI3/AKT-Dependent Autophagy Signaling by Stavudine (D4T) in PBMC of Alzheimer’s Disease Patients

**DOI:** 10.3390/cells11142180

**Published:** 2022-07-12

**Authors:** Francesca La Rosa, Chiara Paola Zoia, Chiara Bazzini, Alessandra Bolognini, Marina Saresella, Elisa Conti, Carlo Ferrarese, Federica Piancone, Ivana Marventano, Daniela Galimberti, Chiara Fenoglio, Elio Scarpini, Mario Clerici

**Affiliations:** 1 IRCCS Fondazione Don Carlo Gnocchi, 20148 Milano, Italy; msaresella@dongnocchi.it (M.S.); fpiancone@dongnocchi.it (F.P.); imarventano@dongnocchi.it (I.M.); mclerici@dongnocchi.it (M.C.); 2 Neurobiology Laboratory, School of Medicine, University of Study of Milano-Bicocca, 20900 Monza, Italy; chiarapaola.zoia@unimib.it (C.P.Z.); chiara.bazzini@unimib.it (C.B.); a.bolognini3@campus.unimib.it (A.B.); elisa.conti@unimib.it (E.C.); carlo.ferrarese@unimib.it (C.F.); 3 Milan Center for Neuroscience, University of Study of Milano-Bicocca, 20126 Milano, Italy; 4 Department of Neuroscience, S. Gerardo Hospital, 20900 Monza, Italy; 5 Fondazione Cà Granda, IRCCS Ospedale Maggiore Policlinico, 20122 Milan, Italy; daniela.galimberti@unimi.it (D.G.); elio.scarpini@unimi.it (E.S.); 6 Department of Biomedical, Surgical and Dental Sciences, University of Milan, 20100 Milan, Italy; 7 Department of Pathophysiology and Transplantation, University of Milan, 20122 Milan, Italy; chiara.fenoglio@unimi.it

**Keywords:** amyloid-β, NLRP3-inflammasome, neuroinflammation, ERK, p38, p70S6K and CREB phosphorylation, caspase-3 and Bcl2

## Abstract

Background: Aβ_42_ deposition plays a pivotal role in AD pathogenesis by inducing the activation of microglial cells and neuroinflammation. This process is antagonized by microglia-mediated clearance of Aβ plaques. Activation of the NLRP3 inflammasome is involved in neuroinflammation and in the impairments of Aβ-plaque clearance. On the other hand, stavudine (D4T) downregulates the NLRP3 inflammasome and stimulates autophagy-mediated Aβ-clearing in a THP-1-derived macrophages. Methods: We explored the effect of D4T on Aβ autophagy in PBMC from AD patients that were primed with LPS and stimulated with Aβ oligomers in the absence/presence of D4T. We analyzed the NLRP3 activity by measuring NLRP3-ASC complex formation by AMNIS FlowSight and pro-inflammatory cytokine (IL-1β, IL-18 and Caspase-1) production by ELISA. The phosphorylation status of p38, ERK, AKT, p70, and the protein expression of CREB, LAMP2A, beclin-1, Caspase-3 and Bcl2 were analyzed by Western blot. Results: Data showed that D4T: (1) downregulates NLRP3 inflammasome activation and the production of down-stream pro-inflammatory cytokines in PBMC; (2) stimulates the phosphorylation of AKT, ERK and p70 as well as LAMP2A, beclin-1 and Bcl2 expression and reduces Caspase-3 expression, suggesting an effect of this compound on autophagy; (3) increases phospho-CREB, which is a downstream target of p-ERK and p-AKT, inducing anti-inflammatory cytokine production and resulting in a possible decrease of Aβ-mediated cytotoxicity; and (4) reduces the phosphorylation of p38, a protein involved in the production of pro-inflammatory cytokines and tau hyperphosphorylation. Conclusions: D4T reduces the activation of the NLRP3 inflammasome, and it might stimulate autophagy as well as the molecular mechanism that modulates Aβ cytotoxicity, and D4T might reduce inflammation in the cells of AD patients. It could be very interesting to check the possible beneficial effects of D4T in the clinical scenario.

## 1. Introduction

Alzheimer’s disease (AD), the most prevalent type of dementia, is characterized by the deposition of amyloid-β (Aβ), the formation of neurofibrillary tangles and neuroinflammation [1]. In AD patients, Aβ accumulation induces the release of inflammatory mediators by microglia; this facilitates Aβ deposition and neuroinflammation in a self-feeding pathogenic loop [2]. On the other hand, microglia play an important role against AD that is mediated by the generation of Aβ-specific antibodies, the clearance of amyloid plaques, and the recruitment of peripheral immune cells that cross the blood–brain barrier (BBB) in an attempt to remove Aβ aggregates [3,4,5]. The initiation of the inflammatory response by microglia and peripheral immune cells involves cytosolic multiprotein platforms known as inflammasomes. The NLRP3 inflammasome is the best characterized of such platforms and its formation requires multiple steps. In a priming step, transcriptionally active signaling receptors induce the NF-kB-dependent induction of *NLRP3* itself, as well as that of the Caspase-1 substrates pro-IL-1β and IL-18 [6]; a second signal leads to the assembly of a multimolecular complex with ASC and Caspase-1. At this point: (i) procaspase-1 or procaspase-8 is recruited [7]; (ii) ASC specks are formed [8]; (iii) caspases are activated; and (iv) cytokines are produced. Activation of the NLRP3 inflammasome is a tightly regulated process. In particular, autophagy, an adaptive response to stress, downregulates NLRP3 inflammasome activation by a complex four metabolic mechanism [9] that includes different molecular pathways [10,11,12,13,14]. Notably, autophagy defects in myeloid cells result in the aberrant activation of the inflammasome [15,16], leading to the development of inflammatory disorders. Different compounds can modulate NLRP3 inflammasome activation. For instance, stavudine (D4T), a nucleoside reverse transcriptase inhibitor (NRTI), was shown to hamper NLRP3 inflammasome activation as well as Caspase-1 and IL-18 production both in vivo [17,18] and in a THP-1-derived macrophage cell line [19]. Notably, in this model, D4T did not have any effect on Aβ phagocytosis, but it stimulated autophagy-mediated Aβ-clearing [19], as witnessed by its ability to modulate ERK1/2 and AKT phosphorylation (p-ERK, p-AKT) and to upregulate LAMP-2A and phospho-p70-S6K, their downstream targets. Given that: (1) autophagy is necessary for the degradation of Aβ in microglia, (2) p-AKT [20] and p-ERK [21] associate with Aβ-accumulation and tau phosphorylation in AD animal models [22,23], and (3) autophagy is modulated by D4T [19], we aimed to explore in more detail the mechanisms responsible for the D4T-mediated autophagy clearing of Aβ. For this aim, we tested D4T involvement in NLRP3 assembly and activation and its possible effect on autophagy molecular signaling in peripheral blood mononuclear cells from AD patients.

## 2. Materials and Methods

### 2.1. Patients

Thirteen AD patients who fulfilled inclusion criteria for a clinical diagnosis of AD were randomly selected within a large database of patients consecutively admitted between January 2017 and September 2019 by the Fondazione IRCCS Ca' Granda Ospedale Maggiore Policlinico in Milano, Italy. All patients were enrolled in a cognitive rehabilitation experimental program and underwent complete medical and neurological evaluation, laboratory analysis, CT scan or MRI, and other investigations (e.g., EEG, SPET scan, CSF examination, etc.) to exclude reversible causes of dementia. The clinical diagnosis of AD was performed according to the NINCDS-ADRDA work group criteria [24] and the DMS IV-R (American Psychiatric Association) [25]. Neuropsychological evaluation and psychometric assessment were performed with a Neuropsychological Battery that included Mini Mental State Examination (MMSE) [26], Digit Span Forward and Backward, Logical Memory and Paired Associated Words Tests, Token Test, supra Span Corsi Block Tapping Test, Verbal Fluency Tasks, Raven Colored Matrices, Rey Complex Figure, and Clinical Dementia Rating Scale (CDR) [27]. The study conformed to the ethical principles of the Helsinki Declaration.

All patients underwent lumbar puncture (LP) for quantification of Aβ, total tau (Τ-τ), and tau phosphorylated at position 181 (P-τ) in the CSF. Normality cut-off thresholds were: Aβ ≥ 600 pg/mL; τ ≤ 500 pg/mL for individuals older than 70 and ≤450 pg/mL for individuals aged between 50 and 70 years; P-τ ≤ 61 pg/mL. The Institutional Review Board of the Fondazione Cà Granda, IRCCS Ospedale Maggiore Policlinico (Milan, Italy) approved this study. All patients (or their caregivers) gave their written informed consent for this research before entering the study.

### 2.2. APO ε4 Genotyping

Apoε genotype was determined by allelic discrimination, as previously described [28]. APOE genotyping was available for all subjects and was dichotomized as being a carrier of at least one ε4 allele or carrying no ε4 alleles.

### 2.3. CSF Collection and Aβ and Tau Determination

CSF was collected between 8 and 10 a.m. after one-night fasting by LP in the L3/L4 or L4/L5 interspace according to standardized local procedures. CSF samples were centrifuged at 2000× *g* for 10 min at 4 °C. The supernatants were aliquoted in polypropylene tubes and stored at −80 °C. CSF cells, glucose, and proteins were determined. CSF Aβ, tau, and P tau were measured using commercially available sandwich enzyme-linked immunosorbent assay (ELISA) kits (Fujirebio, Ghent, Belgium).

### 2.4. Blood Sample Collection and Processing

Whole blood was collected in vacutainer tubes containing ethylenediaminetetraacetic acid (EDTA) (Becton Dickinson & Co., Rutherford, NJ, USA). Peripheral blood mononuclear cells (PBMC) were separated on lympholyte separation medium (Cedarlane, Hornby, ON, Canada) and washed twice in PBS at 2000× *g* for 10 min; viable leukocytes were determined using a TC20 Automated Cell Counter (Biorad Hercules, CA, USA).

### 2.5. Cell Cultures

PBMC from AD patients were resuspended in RPMI 1640 (PAN-Biotech GmbH, Am Gewerbepark, Germany) supplemented with 10% human serum, L-glutamine (2 mM), and 1% penicillin (Invitrogen, Ltd., Paisley, UK) in 6-well plates and incubated at 37 °C in a humidified 5% CO_2_ atmosphere. Cells (7 × 10^6^/well) were primed with Lipopolysaccharide (LPS) (1 μg/mL) (Sigma-Aldrich, St. Louis, MO, USA) for 2 h and stimulated with Aβ_42_ (10 μg/mL Sigma-Aldrich, St. Louis, MO, USA) for 22 h in the absence (untreated)/presence of D4T (50 μM) (Sigma-Aldrich) [29].

### 2.6. MTT Stavudine (D4T)

The MTT assay showed that, in accordance with previous studies [30], PBMC vitality was 90 ± 3.5% using D4T at a 50 µM concentration.

### 2.7. Enzyme-Linked Immunosorbent Assay (ELISA)

IL-1β, IL-18, and activated Caspase-1 production was analyzed in supernatants of PBMC that were resuspended in medium alone or were stimulated (see above) by sandwich immunoassays according to the manufacturer’s instructions (Quantikine Immunoassay; R&D Systems, Minneapolis, MN, USA).

### 2.8. Image Stream Analysis by FlowSight AMNIS

PBMC (2 × 10^6^), stimulated as described above, were fixed with 100 μL of PFA (1%) (BDH, UK), permeabilized with 100 μL of Saponine (0.1%) (Life Science VWR, Lutterworth, Leicestershire, UK), and stained with FITC-anti human NLRP3 (Clone #768319, isotype Rat IgG2a, R&D Systems, Minneapolis, MN, USA) and PE-anti human ASC (clone HASC-71, isotype mouse IgG1, Biolegend, San Diego, CA, USA) for 1 h at room temperature; cells were then washed with PBS, centrifuged at 1500 rpm for 10 min, resuspended in 50 μL of PBS, and examined using the AMNIS FlowSight. Results were analyzed by IDEAS analysis software (Amnis Corporation, Seattle, WA, USA). The analysis of NLRP3 expression was performed by internalization feature, utilizing a mask that represents the whole cell defined by the brightfield (BF) image and an internal mask defined by eroding the whole cell mask. Apoptosis-associated speck-like-protein containing CARD speck formation was analyzed using the same mask of internalization feature, differentiating diffuse or spot (speck) fluorescence inside of cells. Threshold mask was used to separate all ASC positive cells population in ASC-speck spot cells or ASC-diffuse cells by the different diameter of the spot area: in ASC-speck, the spot shows a small area and high max pixel, and the opposite occurs in cells with ASC-diffuse.

### 2.9. Protein Extraction

Cytosol protein extraction was performed in cultured PBMC by Cell Extraction Buffer (BioSource, Thermo Fisher Scientific, Waltham, MA, USA) containing 1 mM PMSF, and protease and phosphatase inhibitor cocktail (Sigma-Aldrich, St. Louis, Missouri, USA) (1:200 and 1:100). After incubation for 30 min on ice, lysates were centrifuged at 12,000× *g* for 10 min at 4 °C and the pellets were discarded. Protein concentration was determined by Bradford assay at 595 nm.

### 2.10. Western Blot

Proteins (15μg) were separated by electrophoresis into 4–12% NuPAGE^®^ Bis-Tris gels (Life Technologies-Invitrogen, Carlsbad, CA, USA) and blotted on nitrocellulose filter (GE Healthcare-Life Sci, Milan, Italy). Blots were blocked 1 h at room temperature on a shaker in 5% fat-free dried milk in TBS-T buffer (50 mM Tris-HCl pH 7.6, 200 mM NaCl, 0.1% Tween 20). Blots were incubated overnight on a shaker at 4 °C with the following rabbit antibody (Ab): anti-p-p38 (T180/Y182 1:350, Cell Signaling, Danvers, MA, USA), anti-p-ERK1/2 (T202/Y204 1:300, Cell-Signaling, Danvers, MA, USA), anti-p-AKT (S473 1:500, Cell Signaling Danvers, MA, USA), anti-p-p70S6K (1:500 Cell Signaling, Danvers, MA, USA), anti-p-CREB (1:800, Biosource, Thermo Fisher Scientific, Waltham, MA, USA), anti-beclin1 (1:300, Cell Signaling, Danvers, MA, USA), anti-LAMP2A (1:400, Abcam, Cambridge, UK), anti-Caspase-3 (1:1000, Sigma-Aldrich, St. Louis, MO, USA), and anti-Bcl2 (1:1000, Sigma-Aldrich, St. Louis, MO, USA). Abs were diluted in 5% fat-free dried milk in TBS-T buffer. A mouse anti-actin Ab (1:20,000, Sigma-Aldrich, St. Louis, MO, USA) was used as standard to normalize the signals of each target protein. Similar data were obtained using specific Abs corresponding to each kinase total protein. A peroxidase-linked anti-rabbit/mouse (1:5000; Sigma-Aldrich, St. Louis, MO, USA) IgG secondary Ab was incubated for 1 h at room temperature on an orbital shaker in TBS-T buffer containing 3% fat-free dried milk. Ten protein samples were run for the different targets. Signals were detected by chemiluminescent reagents (ECL Plus Kit; Amersham, Little Chalfont, Amersham, UK), visualized with a CCD camera using ImageQuant 800 system (ECL Plus Kit; Amersham, Little Chalfont, Amersham, UK), which was quantified using Image-J software, and expressed as the ratio between the target and the actin signals.

### 2.11. Statistical Analysis

Quantitative data were not normally distributed (Shapiro–Wilk test) and are thus summarized as median and interquartile range. Comparisons between the different culture conditions were made using: (1) a 2-tailed Mann–Whitney U test performed for independent samples for NLRP3 complex, cytokine, and caspase production quantified by ELISA; (2) paired Student’s *t* test for protein extracts evaluated by WB analysis. Data analysis was performed using the MedCalc statistical package (MedCalc Software bvba, Mariakerke, Belgium) and the values are expressed as percentage means ± SD; p values less than 0.05 were considered statistically significant.

## 3. Results

### 3.1. Patient Characteristics

The epidemiological, clinical, and genetic characteristics of the AD patients enrolled in the study are presented in Table 1. CSF biomarker analysis was analyzed in all patients and included the concentration of Aβ_42__,_ t-tau, and p-tau (i.e., phosphorylated at threonine 181). Each CSF biomarker was dichotomously classified as positive or negative according to validated cut-off values. Cut-off thresholds of normality were: Aβ ≥ 600 pg/mL; tau ≤ 500 pg/mL for individuals older than 70; and *p*-tau ≤ 61 pg/mL [31]. Results are shown in Table 1.

### 3.2. D4T Downregulates NLRP3-Complex Activation and Inflammasome-Derived Cytokines

**Figure 1:**
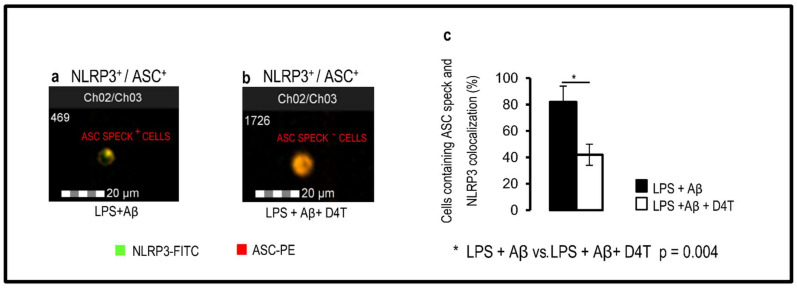
Inflammasome production and ASC-speck formation. Representative images of Nod-like receptor protein 3 (NLRP3) expression and apoptosis-associated speck-like-protein containing CARD (ASC)-speck formation. ASC-speck (**a**) and ASC-diffuse (**b**) images were obtained by AMNIS FlowSight-merged (Ch02/03) NLRP3-FITC (Ch02) and ASC-PE (Ch03) fluorescences; analysis of ASC-speck and its co-localization with NLRP3 was performed by IDEAS software, which provides tools to evaluate image regions (masks) and perform calculations (features). The percentage of ASC-speck-positive cells is shown in panel (**c**). Statistical significance is shown.

**Figure 2:**
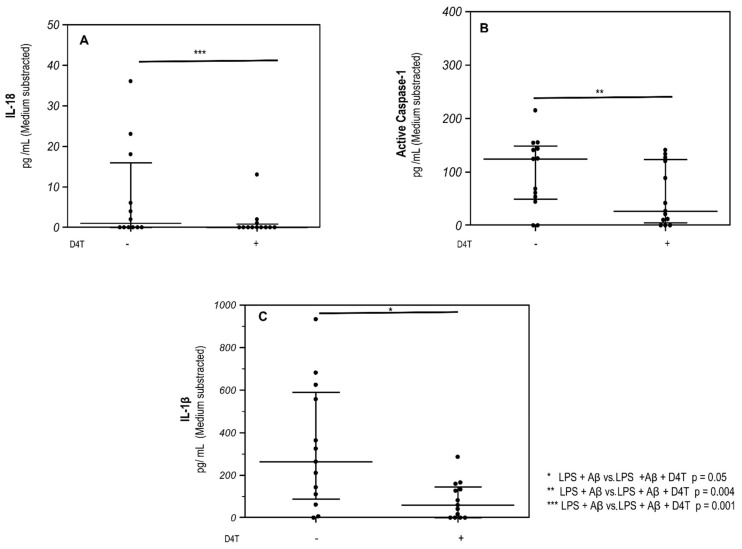
Caspase-1 and inflammasome effector cytokine production. Interleukin (IL)-1β (**A**), Caspase-1 (**B**) and IL-18 (**C**) production by LPS + Aβ-stimulated cells in the presence/absence of D4T were quantified by ELISA. Data are expressed as median cytokine and Caspase-1 concentrations (pg/mL) of stimulated cells with medium subtracted. Statistical significance is shown.

Results obtained by AMNIS FlowSight, when NLRP3 and apoptosis-associated speck-like-protein containing CARD (ASC)-speck formation was investigated showed that in the PBMC of AD patients, D4T prevented the generation of ASC-specks impeding the assembly of NLRP3-inflammasome complexes (Figure 1a,b). Data are shown as a percentage of cells that co-localize ASC and NLRP3-positive cells in which fully functional NLRP3 inflammasome complex formation was significantly reduced (*p* = 0.04) in LPS + Aβ42-stimulated cells in the presence of D4T (Figure 1c). These results were confirmed when activated Caspase-1, IL-18, and IL-1β production was analyzed by ELISA in supernatants collected by cultured PBMC of AD patients. Thus, the production of all these proteins was reduced by D4T (Figure 2a–c); the differences reached statistical significance for IL-18 (*p* = 0.004), activated Caspase-1 (*p* = 0.001), and IL-1β (*p* = 0.05), which are shown in Figure 2a–c.

### 3.3. D4T Modulates MEK-(ERK and p38) and PI3/AKT-Pathways

**Figure 3:**
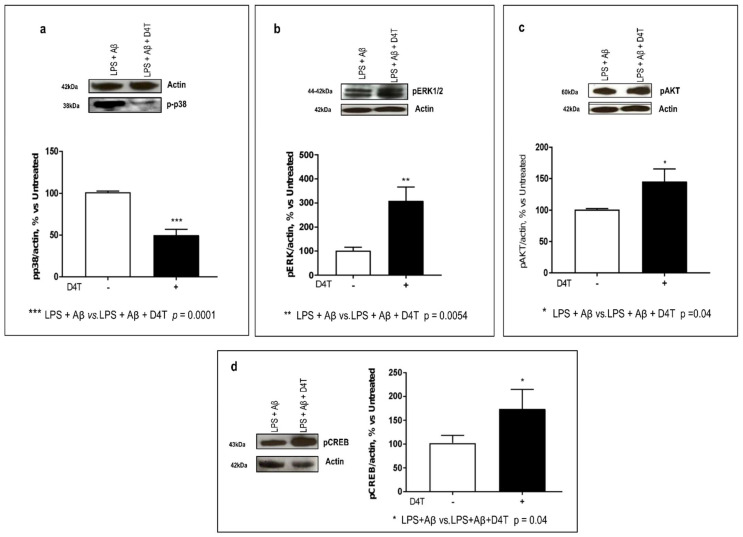
ERK1/2, p38, AKT, and CREB phosphorylation. The phosphorylation status of p38 (38 KDa) (**a**), ERK1/2 (42–44 KDa) (**b**), AKT (60 KDa) (**c**), kinases and CREB (43 KDa) (**d**) were investigated by Western blot analysis (WB) and each result was expressed as the percentage of ratio between phosphorylated-kinase and actin expression versus D4T-untreated cells (100%). Statistical significance is shown as (*** *p* < 0.0001, ** *p* < 0.001, * *p* < 0.05). Representative blots from independent experiments are shown in the upper part of panels (**a**–**d**).

The NLRP3 inflammasome can also regulate autophagy by modulating the expression of phosphatases and kinases. By WB, the ERK, p38, and AKT phosphorylation was analyzed in LPS-primed and Aβ_42_-stimulated-PBMC of AD patients. Our results showed that the D4T treatment to cultured PBMC significantly downmodulated p-p38 (*p* = 0.0001) (Figure 3a), whereas it upregulated p-ERK1,2 (*p* = 0.0054) (Figure 3b) and p-AKT (*p* = 0.04) (Figure 3c).

CREB is also a downstream target in the ERK- and AKT-mediated pathways. Its phosphorylation (p-CREB) status was also investigated, and it was increased following the D4T treatment (*p* = 0.04) (Figure 3d). Additionally, p-CREB also promotes anti-inflammatory cytokines production.

### 3.4. Effect of D4T on Autophagy Signaling

**Figure 4:**
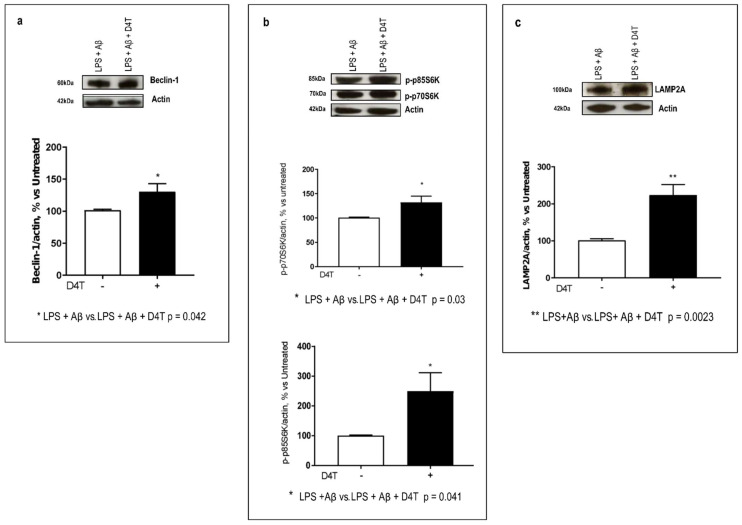
Beclin-1- and CMA-mediated autophagy proteins. Western blot analyses of Beclin-1 ((**a**), 52 KDa) and its downstream targets phospho-p70S6K and phospho-p85S6K ((**b**) 70 and 85 KDa) and Lamp2A ((**c**) 100 KDa), the receptor for CMA, are shown. Each result is expressed as a percentage of the ratio between the protein expression or phosphorylated-kinase and actin expression versus D4T-untreated cells. Statistical significance is shown as (** *p* < 0.001, * *p* < 0.05). Representative blots from independent experiments are shown in the upper part of panels (**a**–**c**).

Interaction of NLRP domains with autophagy proteins seems to provide a mechanism for direct NLRP regulation of autophagy. In particular, in this study, inflammasome-forming NLRP subunits were found to interact with the mTOR/Beclin-1 pathway that is modulated by ERK- and AKT-phosphorylation status. For this reason, we tested the D4T effect on phospho-p70S6K, a downstream target of ERK and AKT pathways that is also involved in autophagy signaling, and its phosphorylation and activity are required for LC3-II formation, too. By WB, Beclin-1 expression, phospho-p70S6K status, and LAMP2A expression were examined. Beclin-1 was slightly increased by D4T (*p* = 0.042, Figure 4a); on the other hand, the phosphorylation of p70S6Kinase was significantly increased by D4T (*p* = 0.03, Figure 4b). Notably, both phospho-p70S6K isoforms, the 70 KDa cytosolic form and the 85 KDa nuclear one, were significantly upregulated by D4T (*p* = 0.04) (Figure 4b) as well as LAMP2A (*p* = 0.0023) (Figure 4c). This is a protein in the AKT-mediated pathway and it is also the receptor for chaperon-mediated autophagy (CMA).

### 3.5. Beclin-1 Regulating Proteins by D4T

**Figure 5:**
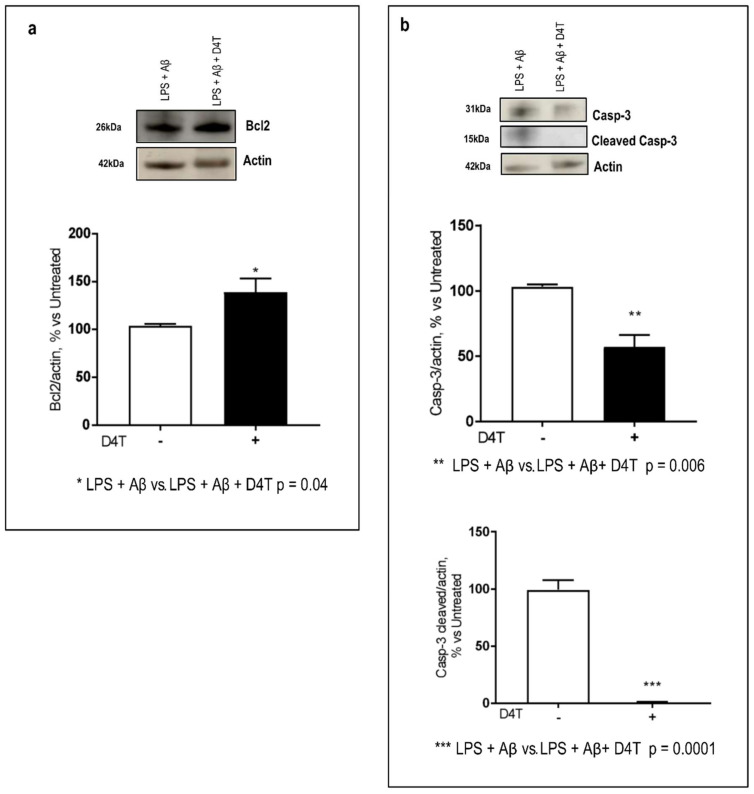
Caspase-3/cleaved-Caspase3 and Bcl2 production. Western blot analysis of Bcl2 ((**a**) 26 KDa) and Caspase-3 ((**b**) 31 KDa) and cleaved Caspase-3 ((**b**) 15 KDa) were evaluated. Each result is expressed as a percentage of the ratio between each target and actin expression versus D4T-untreated cells. Statistical significance is shown as (*** *p* < 0.0001, ** *p* < 0.001, * *p* < 0.05). Representative blots from independent experiments are shown in the panels (**a**,**b**).

Since Beclin-1 is a pivotal protein that regulates macroautophagy and also apoptosis, we investigated the ability of D4T to modulate beclin-related pathways. Many proteins, including Bcl-2 (an anti-autophagy protein), and proteases, such as Caspase-3, can interact with Beclin-1 to regulate its autophagy function; Beclin 1 is a dual regulator for both autophagy and apoptosis. When Caspase-3 is activated (cleaved Caspase-3, 15 kDa), it destroys the binding between Beclin-1 and Bcl-2, and it switches Beclin-1 from a pro-autophagic to a pro-apoptotic protein. By WB, D4T interestingly induces: (1) an increase in Bcl2 (*p* = 0.04, Figure 5a) and (2) a significant reduction of Caspase-3 (*p* = 0.006) and a more significant downregulation of cleaved Caspase-3 (*p* = 0.0001) (Figure 5b).

## 4. Discussion

Microglia-driven neuroinflammation has been identified as a key player in the pathogenesis of AD; the formation of oligomer Aβ_42__,_ in particular, is an early event in the disease that induces microglia activation and inflammation. It has been shown that this process is highly dependent on the activation of the NLRP3/ASC-inflammasome both in microglial cells [32,33] and in circulating peripheral monocytes [34]. Thus, systemic inflammation triggers a neuroinflammatory response that results in microglial activation with deleterious consequences for learning and memory in rodent models [35,36,37] and in humans [38,39,40,41,42,43,44]. According to the literature, neuroinflammation leads to autophagy downregulation [45,46], and we recently reported that NLRP3/ASC-inflammasome activation hampers the microglial clearance of Aβ in an in vitro experimental model of the THP-1-derived macrophage cell line [19]. Notably, in that experimental setting, stavudine (D4T), a common drug used as therapy in HIV-infection, prevented active Caspase-1 release, but it did not restore microglial Aβ phagocytosis. Given this background, we investigated Aβ-stimulated inflammation and autophagy cell signaling and the effects of D4T on these molecular mechanisms in the peripheral blood immune cells of AD patients.

Results herein show that D4T is capable of significantly reducing NLRP3 inflammasome activation and the downstream production of IL-18 and Caspase-1 even in the PBMC of AD patients, but it has only a marginal effect on IL-1β. Recent results also indicate that gasdermin D, a caspase substrate that mediates the activation of NLRP3-independent pyroptosis, induces the formation of pores in the plasma membrane. Because pores can serve as a gate for the release of mature IL-1β from cells [47], this could explain the different behavior of IL-18, Caspase-1, and IL-1β.

Autophagy is an intracellular degradation process responsible for the delivery of damaged organelles and proteins from the cytoplasm and it is involved in programmed cell death and neurodegeneration; furthermore, autophagy also represents an alternative pathway of cellular defense by removing intracellular pathogens [48,49,50]. The three main forms of autophagy are: macroautophagy (mTOR-beclin-1-dependent) and microautophagy (endosomal-mediated and CMA), with the latter being associated with lysosomal degradation; it is mediated by a cytoplasmic complex of chaperone proteins that interacts with LAMP2A. All these three forms of autophagy crosstalk with each other to eliminate aberrant proteins and modulate cell apoptosis, too [51,52,53].

In this work, the autophagy cell signaling was analyzed, and the results show that the phosphorylation status of AKT, ERK, and cytosolic and nuclear p70S6 kinases as well as the expression of LAMP2A and Beclin-1 increase in the D4T-stimulated PBMC of AD patients, indicating that D4T upregulates the pro-autophagic downstream AKT- and MAP-kinase pathways.

Macroautophagy is the best studied form of autophagy, and since the Bcl-2/Beclin-1 interaction seems to function as a key regulatory mechanism in autophagy [54], ability of D4T to regulate this binding. Beclin-1 can be cleaved by Caspase-3 (15 kDa), and this cleavage results in an inactivation of autophagy [55,56]. In this work, D4T induced a significant downregulation of Caspase-3 expression and a complete reduction in its cleaved isoform; further, it exhibited a trend to upregulate Beclin-1 and Bcl-2 In normal conditions, Bcl-2, an anti-apoptotic protein, binds Beclin-1, thus resulting in an inactivation of autophagy; while under stress, Beclin-1 dissociates from Bcl-2 allowing the activation and subsequent stimulation of autophagy [54]. Since it has been shown [55] that the cleavage of Beclin 1 by Caspase-3 may contribute to inactivating autophagy, leading towards augmented apoptosis, our results suggest DT4 decreases Caspase-3 activation, counteracting the autophagy inhibition.

CREB is another downstream mediator in ERK- and AKT-mediated pathways, and it also regulates anti-inflammatory IL-10 production, modulating its phosphorylation status. The D4T-mediated upregulation of p-CREB could stimulate IL-10 production to counteract Aβ toxicity. Moreover, the D4T-induced modulation of CREB is also particularly important as this protein has a well-documented role in neuronal plasticity and long-term memory formation [56,57]; in fact, p-CREB expression is reduced in the PBMC of AD subjects [58,59,60]. In a clinical scenario, D4T might also be beneficial for CNS cells, not only for blood peripheral cells. Notably, behavioral symptoms and progression of AD in a murine model were shown to be alleviated by the activation of the PI3K/AKT signaling pathway [61], which is an upstream pathway of CREB.

The effect of D4T on p38 signaling is potentially relevant, too. Thus, p38 is activated in macrophages, neutrophils, and T cells by extracellular mediators of inflammation including cytokines, chemokines, and LPS [62,63], and phospho-p38 induces the Th1 immune response and the production of pro-inflammatory cytokines [64]. Furthermore, p38 is also involved in tau hyperphosphorylation, and the D4T downregulation of p38 phosphorylation might be also useful to prevent tau phosphorylation and tangle production. The observed D4T downregulation of this protein thus could be beneficial in reducing Aβ oxidative stress and cytotoxicity. Oxidative stress plays a pivotal role in the initiation and progression of neurodegenerative diseases; indeed, recently it was reported that the dual activation of the MAPK/ERK and PI3K/AKT pathways was found to be synergistic to mitigate the cytotoxicity induced by aggressive oxidative stress. Currently, a wide range of small molecule inhibitors have been proposed for the simultaneous targeting of the MAPK/ERK and PI3K/AKT signaling pathways, which may be effective in a treatment to prevent oxidative-stress-related diseases [65].

## 5. Conclusions

Efficient autophagic activity prevents the activation of inflammasomes [66] and the stimulation of autophagy was shown to reduce soluble Tau as well as Aβ and amyloid plaques in 3xgAD mice [67]. On the other hand, it is known that Beclin 1-dependent autophagy is downregulated in AD [68] and the NLRP3 inflammasome is activated in the brain and PBMC of AD patients [34,69]. In this manuscript, we show the results obtained in the peripheral immune cells of AD patients that indicate the D4T involvement in reducing NLRP3 inflammasome activation, upregulating autophagy as well as inhibiting anti-inflammatory signaling. Although a limitation of the present work is the sample size, taken together, these findings support the hypothesis that the NLRP3 inflammasome may be a potential therapeutic target for and suggest that D4T is a possible rehabilitation treatment for this disease, thus warranting future investigations into the use of D4T in the clinical scenario.

## Figures and Tables

**Table 1:** Demographic, clinical, and genetic characteristics of the individuals enrolled in the study.

	Alzheimer’s Disease Patients (AD)
N	13
Gender (M:F)	5:8
Age (years)	77.20 ± 6.34
MMSE	17.07 ± 4.51
Aβ (pg/mL)	530 ± 70.50
Total-τ (pg/mL)	647.31 ± 300
Phospo-τ (pg/mL)	88.62 ± 21.89
Apo ε4	30%

Data are expressed as mean ± standard deviation. MMSE: Mini-Mental State Examination.

## Data Availability

The datasets used and/or analyzed during the current study are available from the corresponding author on reasonable request.
